# Interactive visual exploration of overlapping similar structures for three-dimensional microscope images

**DOI:** 10.1186/s12859-014-0415-x

**Published:** 2014-12-19

**Authors:** Megumi Nakao, Shintaro Takemoto, Tadao Sugiura, Kazuaki Sawada, Ryosuke Kawakami, Tomomi Nemoto, Tetsuya Matsuda

**Affiliations:** Graduate School of Informatics, Kyoto University, Yoshida Honmachi, Sakyo, Kyoto Japan; Graduate School of Information Science, Nara Institute of Science and Technology, 8916-5, Takayama, Ikoma, Nara Japan; Graduate School of Information Science and Technology, Hokkaido University, Sapporo, Hokkaido Japan; Research Institute for Electronic Science, Hokkaido University, Sapporo, Japan

**Keywords:** Interactive visualization, Multi-dimensional transfer functions, Neural structures, Microscopic images

## Abstract

**Background:**

Recent advances in microscopy enable the acquisition of large numbers of tomographic images from living tissues. Three-dimensional microscope images are often displayed with volume rendering by adjusting the transfer functions. However, because the emissions from fluorescent materials and the optical properties based on point spread functions affect the imaging results, the intensity value can differ locally, even in the same structure. Further, images obtained from brain tissues contain a variety of neural structures such as dendrites and axons with complex crossings and overlapping linear structures. In these cases, the transfer functions previously used fail to optimize image generation, making it difficult to explore the connectivity of these tissues.

**Results:**

This paper proposes an interactive visual exploration method by which the transfer functions are modified locally and interactively based on multidimensional features in the images. A direct editing interface is also provided to specify both the target region and structures with characteristic features, where all manual operations can be performed on the rendered image. This method is demonstrated using two-photon microscope images acquired from living mice, and is shown to be an effective method for interactive visual exploration of overlapping similar structures.

**Conclusions:**

An interactive visualization method was introduced for local improvement of visualization by volume rendering in two-photon microscope images containing regions in which linear nerve structures crisscross in a complex manner. The proposed method is characterized by the localized multidimensional transfer function and interface where the parameters can be determined by the user to suit their particular visualization requirements.

## Background

Given the complex three-dimensional (3D) architecture of the brain, it is essential to explore the morphology and activity of neurons in all layers of the cortex. However, this can often be challenging because the dendrites of cortical neurons are widely spread across several layers, including deeper layers that are difficult to observe by confocal or light microscopy. The length of the dendrites can vary from 20 μm to 1 mm, and the width and branching of the dendrites depends on the distance from the soma. This suggests that spatial differences in brain morphology relate to the functionality of the neuron, including characteristics of the dendrite and synaptic efficiency [1,2].

To understand the 3D structure of dendrites and the connectivity among neurons in the brain, in vivo imaging and visualization have significant roles. Recently, the improved performance of microscopy systems has enabled the acquisition of large amounts of slice images from living tissues. In comparison with confocal or other optical microscopy systems, two-photon microscopy has an advantage in visualizing the morphology of neurons within deeper layers of a living mouse brain [3-6]. Because the structures of tissues are stored as volume data, volume visualization techniques [7-9] are focused on the interactive exploration of the 3D images. When visualizing unknown features in the deeper layers of the brain, prior knowledge of the morphology of tissues [10,11] cannot be used. Furthermore, microscopic images are affected by optical characteristics such as scattering within tissues and the presence of image noise within the deeper regions of the images. Because large amounts of volume data are obtained through two-photon microscopy, there is a demand for efficient visualization of local internal structures and characteristic intensity distributions.

Direct volume rendering (DVR) has been widely used for visualizing volume data, where the rendered image is generated from the data by simulating optical properties such as radiation and absorption [12,13]. The user can interactively explore the micro-level structures included in the volume data while changing the camera parameters, modifying the transfer functions [14-16] or generating the cross-section of the 3D images. Unlike the pattern recognition approach [17-19], visualization does not involve algorithm-based detection for specific objects. In other words, the only aspects defined by the method are the transformations when visualizing the 3D image as a projection on the screen, and modifications of the visualization parameters and final judgments about the structures observed are left to the user.

The final quality of projections obtained with volume rendering depends significantly on the definition of the transfer functions (TFs). For this reason, the design of the TFs is regarded as an important area of research for volume visualization. Clinical tools commonly provide predefined TFs (TF presets) for efficiency in the clinical workflow. Recent studies have improved the TFs based on multidimensional feature values to visualize changes in texture and morphological characteristics included in the images [20-24]. Because the high degree of freedom in multidimensional TFs makes it difficult for users to obtain visualization results through manual parameter settings, a variety of user interfaces and automatic TF generation methods have been investigated [25-27]. In the case of visual exploration, there are many situations for which feature descriptors have not been formulated [28]. Some researchers have focused on this issue and have investigated methods for exploring high-dimensional feature space. Principal component analysis, independent component analysis, and clustering techniques are commonly used for dimensionality reduction of the feature space [29,30].

**Figure 1:**
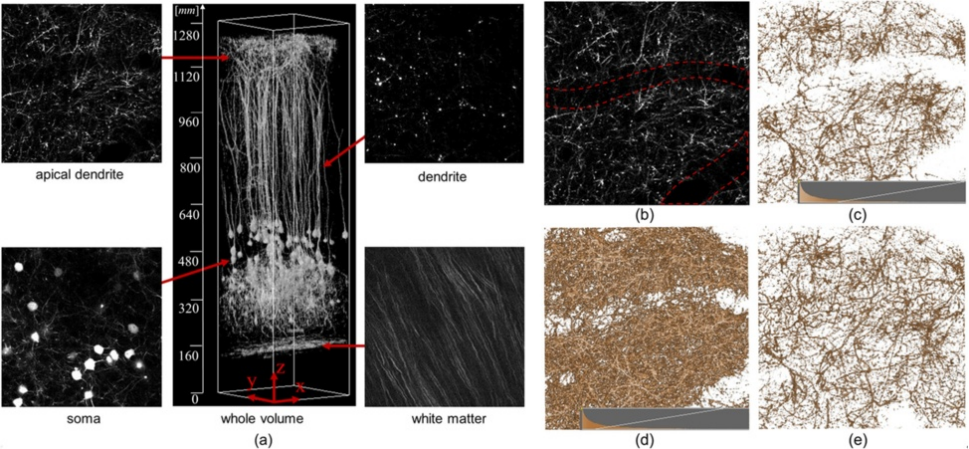
**Visual exploration of overlapping neural structures.** **(a)** An example of three-dimensional microscope images with a depth ~1.3 mm from the surface layer of the cortex. **(b)** Tomographic image of apical dendrites. The low contrast area shadowed by the blood vessels is shown within the red dashed lines. Volume visualization results obtained by **(c) (d)** a traditional intensity-based 1D TFs and **(e)** the proposed method, which succeeds in locally improving the visualization result.

In microscope images, the intensity value of voxels can differ locally, even within the same structure, because emission of fluorescent materials and the optical properties based on point spread functions affect the imaging results. Most previous TFs designed for clinical computed tomography (CT) and magnetic resonance imaging (MRI) images are not applicable to images that have specific areas with locally varying contrast. In addition, images obtained from brain tissue contain a variety of neural structures such as dendrites and white matter, where similar line structures are overlapped or crisscrossed with some complexity (see Figure 1). Numerous minute or thin structures can be closely observed with optical scattering noise. In these cases, previous TFs often fail to optimize the visualization results, making it difficult to explore the connectivity of neurons because of their occlusions and geometric complexity. The main focus of this study, therefore, is to answer these issues in biological visualization, and to design an efficient visual exploration tool.

This paper proposes an interactive visual exploration method by which the TFs are modified locally and interactively based on multidimensional features of the images. This method will also provide a direct editing interface to specify both the target region and the structures with characteristic features, where all manual operations can be performed on the rendered image. Therefore, multidimensional features and interactive methods can be used for local improvement of the visualization results for overlapping structures. Despite the high dimensionality of the TFs, users only specify the structures of interest on the rendered image and control simple parameters to explore similar structures. The performance of the method is demonstrated using two-photon microscope images measured from live mice. The experiments show that this method is effective for interactive visual exploration of overlapping similar structures.

Regarding related study in vessel visualization, the recent work by Kubisch et al. [31] summarizes problems and reviews of related work. Much recent work has focused on multi-scale methods based on eigenvalue analysis of the Hessian [18]. Using the vesselness measure, Lathen et al. [27] presented automatic tuning techniques by applying a locally shifted intensity to the TFs (called a TF shift) in the vessel visualization domain. In computed tomography angiography (CTA), which is analogous to microscope images, the intensity value of blood vessels partially decrease owing to the distribution deflection of the contrast medium. The advantage of this approach is to use the 1D TF presets that are popular in clinical applications and are easily set up in a visualization workflow.

In this study, we consider an application of the TF shift technique to 3D microscope images. In biological visualization using two-photon or confocal microscope images, however, the main focus is on neural structures that crisscross in a complex way with multiple overlaps. The deep layers of the brain tissue contain a variety of neural structures with complex shapes such as soma, dendrites, and white matter. Unlike the situation that exists in clinical CT or MRI images, however, numerous minute or thin structures are closely observed with optical scattering noise, which creates challenges for volume visualization. To our knowledge there have been no reports on an interactive, visual exploration software and interface for overlapping similar neural structures in microscope images. This study proposes a new TF shift mechanism and interface that can efficiently use more general multidimensional feature values, while avoiding a complex TF design process.

## Methods

Figure 1 shows slice images that include structures with characteristic shapes measured from a live mouse using a two-photon microscope. The central image shows the results of visualization of the entire image data with an intensity-based 1D TF. Apical dendrites, dendrites, soma, and white matter, which are all parts of the neurons, are included in the images. In the apical dendritic region, linear structures run along the *xy* plane and each linear structure crisscrosses in a complex manner. The large linear shadow shown within the red frame in the middle of the image indicates blood vessels, because there is a tendency for a decrease in the intensity value of structures directly below blood vessels. In the dendritic regions, linear structures with a high intensity value can be found along the *z* axis and, around these, linear structures with low intensity values exist in large quantities, though these structures are not visible. The soma can be identified as a spherical structure. In the white matter region, most of the linear structures run in the specific direction of the *xy* plane.

We will now discuss our goals toward visualization in the microscope images. Figure 1(b) is a tomographic image of apical dendrites located near the brain surface. However, there is actually a large quantity of low luminance neurons around the dendrites, making observation of the dendrites difficult. Blood vessels with a diameter of ~30 voxels run within this data, and are seen as regions in which nerves do not exist. The low contrast area shadowed by the blood vessels is shown within the red frame in Figure 1(b). Figure 1(c) and (d) shows the volume visualization results obtained by a traditional intensity-based 1D TF. The histogram of the voxel and the opacity curve setting in the TF is inserted in the bottom of each figure. In Figure 1(c), it is not possible to distinguish most of the structures with low intensity under the area of blood vessels. Figure 1(d) shows another visualization result obtained by adjusting the window level parameter of the TF to a lower level. In this figure, some structures can be observed in the low contrast area. However, because of a widening in the range of opaque intensity, surrounding dendric structures and optical noises can occlude the target structure, which fails to distinguish the connectivity of the three-dimensional neural networks. Figure 1(e) is a visualization result obtained by the method proposed in this study, which succeeds in distinguishing dendric structures and their connectivity under the blood vessels. Because the color/opacity outside the target area does not change, the target structures with low intensity as well as the other structures outside of the region of interest can be seen simultaneously without self-occlusion or noise enhancement.

Here, we describe a set of methods to achieve the above-mentioned local refinement of the rendered image. Because intensity values and contrast can differ locally in the target data of this study, the method developed by Lathen et al. for CTA images [27] is used as a basis for the local adjustment of TFs. To achieve automatic tuning of visualization results for linear structures, Lathen et al. employed the *vesselness* measure as feature values. In our study, for visualization of various neural structures such as soma, dendrites, and apical dendrites with different features and sizes, we design a novel TF shift framework to address these multidimensional feature values. In addition, for efficient visualization of dendric structures that crisscross in a complex manner, locally similar structures are selectively visualized by direct editing of the visualization results on the rendered image.

**Figure 2:**
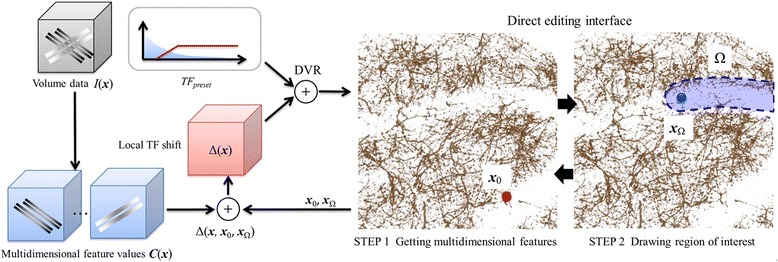
**Flowchart and user interface of the proposed visualization framework.** Multidimensional feature values of microscope images are addressed in visualization process with local TF shift. With the direct editing interface, the user can explore overlapping similar structures with spatially different contrast locally and interactively.

Flowcharts of the proposed method are shown in Figure 2. With the proposed method, a predefined 1D TF (*TF*_*preset*_) is first prepared, and DVR of the target data is conducted. The colored histogram with a linear graph at the bottom of Figure 1(c) and (d) is used as a TF setting interface, which is a common interface in medical visualization software. The user can change the color lookup table that maps the intensity to RGBA values on the histogram. Opacity values are defined using the linear graph or using free form curves manually drawn by the user. These configurations can be done by mouse operation only. In addition to modifying TFs, users can obtain desired visualization results based on the rendered images. Users can directly specify the structures of interest Ω ∈*R*^3^ as visualization targets. During visualization workflow, users input into the system one point of a structure (***x***_0_) similar to the structures of interest such that ***x***_0_ ∈*R*^3^, and the central coordinate of differential Ω (***x***_Ω_). Our system calculates the shift value (Δ) of the TF of all voxel positions (*x*) within Ω on the basis of ***x***_Ω_ and ***x***_0_. Then, the input for the TF is modified, and DVR is again processed. If users are not satisfied with the rendering results, the TF can be modified again.

**Figure 3:**
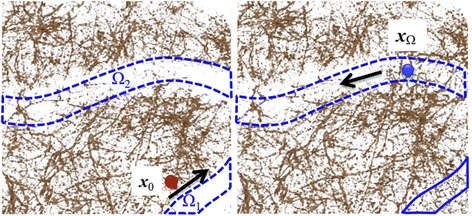
**Interactive editing of the visualization results.** By locally changing the shift value of the TF in the low-luminance area, similar structures with low intensity values can be visualized based on multidimensional features.

Figure 3 shows changes in visualization when ***x***_0_ is fixed and Ω is altered, along with the shift value Δ of the TF. In this case, the shape of the 3D pointer is a sphere, which works as a minimal ROI for the local TF shift. The radius of the pointer is changed by the user, and the time series input by dragging a mouse defines the total ROI Ω. Therefore, the shape of the ROI can be modified interactively and can be any form. By increasing the shift value of the TF in low-intensity voxels, if there is a similar structure one can assume the visualization results to be the same, regardless of the intensity value. The TF configured by the user is stored in a file to be re-used by different users or the same user at different times. Because the TFs consist of volumetric, spatially-varying scalar values, the file format can be the same as three-dimensional image format.

### Localized multidimensional transfer function

This section describes details of the designed spatially varying TF based on multidimensional feature values. In a method similar to the approach used by Lathen et al. [27], our system uses a basic objective function based on the Eq. (1)

1 $$ TF\left(\boldsymbol{x},\ I\left(\boldsymbol{x}\right),\Delta \left(\boldsymbol{x}\right)\right) = T{F}_{preset}\left(I\left(\boldsymbol{x}\right) + \Delta \left(\boldsymbol{x}\right)\right), $$

where *I* is the intensity distribution of the volume data.

For selective visualization of a variety of structures contained in a microscope image, the design of the calculation formula of the shift value Δ of the TF becomes important. Specifically, unlike the previous TF shift technique based on the *vesselness* measure, multidimensional features are addressed in our framework. The following section explains the concrete calculation formula of Δ. In addition, to allow users to interactively input ***x***_0_ and ***x***_Ω_ into the system, it is essential to develop a method for inputting 3D coordinates on the rendering image.

The calculation formula of Δ is designed to meet Conditions (A), (B), and (C), given as

(A) If *I*(***x***) = *I*(***x***_0_), then *RGBA*(***x***) = *RGBA*(***x***_0_).
(B) If ***C***(***x***) ≈ ***C***(***x***_0_), *RGBA*(***x***) ≈ *RGBA*(***x***_0_).
(C) Only *RGBA*(***x***) changes within the region Ω near ***x***_Ω_.

In these conditions, the color/opacity value of point ***x*** is expressed as *RGBA*(***x***), and the feature value of point ***x*** is expressed as ***C***(***x***) ∈*R*^*n*^.

Condition (A) is used to maintain the integrity of the calculation. In the proposed method, reference point ***x***_0_ can also become a processing target. For the situation where there is no shift in the TF of the reference point ***x***_0_, Condition (A) must be feasible. Condition (B) indicates that the color/opacity value of structures similar to ***x***_0_ changes. If the features are similar, even with structures of different luminance, visualization can be achieved on the basis of the color/opacity value previously determined with *TF*_*preset*_. Condition (C) indicates that only the color/opacity value in the region Ω specified by the user changes. Visualization that maintains visibility can be achieved through spatial and local visualization when structures with multiple overlaps are not portrayed. To satisfy Condition (A), the shift function Δ(***x***, ***x***_0_***, x***_Ω_) should have *I*(***x***_0_) − *I*(***x***) as a factor. Introducing the function *g*(***x***, ***x***_0_***, x***_Ω_) where (1 ≥ *g* ≥ 0), Δ is determined as

2 $$ \varDelta \left(\boldsymbol{x},{\boldsymbol{x}}_0,{\boldsymbol{x}}_{\varOmega}\right)=\left(I\left({\boldsymbol{x}}_0\right)-I\left(\boldsymbol{x}\right)\right)g\left(\boldsymbol{x},{\boldsymbol{x}}_0,{\boldsymbol{x}}_{\varOmega}\right). $$

When *g* = 1, then Δ = *I*(***x***_0_) − *I*(***x***) and *TF*_*preset*_ becomes *TF*_*preset*_(*I*(***x***_0_)). This means that the color/opacity value *RGBA*(***x***) in ***x*** becomes the same as *RGBA*(***x***_0_) in ***x***_0_. When *g* = 0, then Δ = 0, and the color/opacity value *RGBA*(***x***) of ***x*** is unchanged. To achieve Condition (B), the dissimilarity of ***C***(***x***) and ***C***(***x***_0_) (*d*_*F*_(***C***(***x***),***C***(***x***_0_)) where (*d*_*F*_ > 0)) in the feature value space is calculated. A larger *d*_*F*_ relates to a greater difference in the features of structures in ***x*** and ***x***_0_. The Mahalanobis distance and inner product can be used in the calculation of *d*_*F*_. Equation (5) shows cases in which the Mahalanobis distance *d*_*mah*_ is used in Eq. (3), and when the inner product *d*_*dot*_ is used in Eq. (4).

3 $$ {d}_{mah}\left(\boldsymbol{C}\left(\boldsymbol{x}\right),\boldsymbol{C}\left({\boldsymbol{x}}_0\right)\right)=\sqrt{{\left(\boldsymbol{C}\left(\boldsymbol{x}\right)-\boldsymbol{C}\left({\boldsymbol{x}}_0\right)\right)}^T{\boldsymbol{V}}^{-1}\left(\boldsymbol{C}\left(\boldsymbol{x}\right)-\boldsymbol{C}\left({\boldsymbol{x}}_0\right)\right)}, $$

4 $$ {d}_{dot}\left(\boldsymbol{C}\left(\boldsymbol{x}\right),\boldsymbol{C}\left({\boldsymbol{x}}_0\right)\right)=1-\boldsymbol{C}\left(\boldsymbol{x}\right)\cdot \boldsymbol{C}\left({\boldsymbol{x}}_0\right), $$

where ***C***(***x***) is the feature value vector at ***x*** and ***V*** is the covariance matrix between every feature value. To achieve Condition (C), the distance of ***x*** and ***x***_Ω_ is calculated in the virtual space by the Euclidean distance *d*_*E*_(***x***,***x***_Ω_) where (*d*_*E*_ > 0). When considering the function *g*(***x***,***x***_0_***,x***_Ω_) that satisfies Conditions (B) and (C), *g* is first defined, as with Eq. (5). Next, the function *f*_*F*_, which changes in value according to the dissimilarity of features, and the function *f*_*E*_, which determines the range of Ω, are introduced as

5 $$ g\left(\boldsymbol{x},{\boldsymbol{x}}_0,{\boldsymbol{x}}_{\varOmega}\right)={f}_F\left({d}_F\left(\boldsymbol{x},{\boldsymbol{x}}_0\right)\right){f}_E\left({d}_E\left(\boldsymbol{x},{\boldsymbol{x}}_{\varOmega}\right)\right). $$

**Figure 4:**
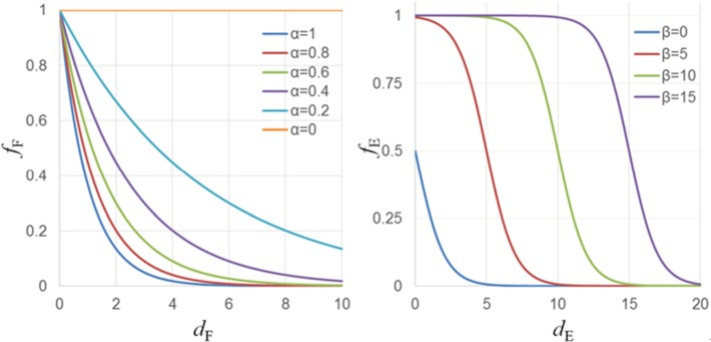
**Two sub-functions** ***f***_***F***_ **and** ***f***_***E***_ **defined by the configurable parameters, where the parameter** ***α*** **is the sensitivity that determines the amount of the change in RGBA dependent on the dissimilarity of the feature values and parameter** ***β*** **is the radius of the current region of Ω.**

To satisfy 1 ≥ *g* ≥ 0, *f*_*F*_ and *f*_*E*_ must each satisfy 1 ≥ *f*_*F*_ ≥0 and 1 ≥ *f*_*E*_ ≥0. Function *f*_*F*_, which satisfies Condition (B), and the sigmoid function *f*_*E*_, which satisfies Condition (C), are shown in Eqs. (6) and (7), respectively. An outline of these functions is given in Figure 4. By using the sigmoid function in *f*_*E*_, the visualization results at the boundaries of Ω can be easily changed, and improvement in visualization quality can be expected.

6 $$ {f}_F\left({d}_F\right)={e}^{-\alpha {d}_F}, $$

7 $$ {f}_E\left({d}_E\right)=\frac{1}{1+{e}^{d_E-\beta }}, $$

where the configurable parameters *α* ≥ 0 and *β* ≥ 0 are introduced by the user. Parameter *α* is the sensitivity that determines the amount of the change in *RGBA* dependent on the dissimilarity of the feature values. Parameter *β* is the radius that determines the size of region Ω. The shift value Δ when *f*_*F*_ and *f*_*E*_ from Eqs. (2), (6), and (7) are used is given as

8 $$ \varDelta \left(\boldsymbol{x},{\boldsymbol{x}}_0,{\boldsymbol{x}}_{\varOmega}\right)=\left(I\left({\boldsymbol{x}}_0\right)-I\left(\boldsymbol{x}\right)\right)\frac{e^{-\alpha {d}_F}}{1+{e}^{d_E-\beta }}. $$

### Direct editing interface

In this section, we consider the method for directly inputting the 3D coordinates ***x***_0_ and ***x***_Ω_ onto the rendered image. With the volume rendering process, volume data are projected in the viewing direction ***ν***_*eye*_∈*R*^3^. As such, there are voxels drawn by the click point (***x***_*click*_∈*R*^2^) of the rendered image in the direction of ***ν***_*eye*_, which originates from ***x***_*click*_. Among the quantity of voxels existing in the direction of ***ν***_*eye*_, those close to the surface of the structure are believed to be the voxels specified by the user and are estimated using degrees of transparency [32,33]. First, the volume data are searched from the ***x***_*click*_ point in the direction of ***ν***_*eye*_. During the search, voxels are sampled at fixed intervals, ***x***_*i*_. Next, the sum ${\displaystyle {\sum}_{i=0}^n}{\alpha}_i$ of the opacity value (*α*_*i*_) of ***x***_*i*_ is calculated. The value of *α*_*i*_ is calculated using *TF*(***x***, *I*(***x***), Δ(***x***)), which considers Δ. The voxel is acquired that is larger than a threshold opacity value (*α*_*th*_) predefined by ${\displaystyle {\sum}_{i=0}^n}{\alpha}_i$, and these voxels are assumed to be those specified by the user and are close to the surface of the structure that has been visualized to consider the Δ.

## Results and discussion

We implement a sequence of algorithms using C++, OpenGL, GLSL (Open GL Shader Language), and the software package NVIDIA CUDA (Compute Unified Device Architecture). We apply the developed software to two-photon microscope images and verify the visualization of the characteristic structures and the intensity distribution included in the images with biological researchers. For the 3D microscope image and for visualization of its feature volume, we use the texture-based rendering scheme [13] to achieve high-speed volume rendering using the texture interpolation and synthesis functions of the graphics processing unit. For verification, we used three volume data sets taken from live, genetically modified mice [4] using a Nikon two-photon microscope (A1MP+), wherein the neurons in the second and fifth layers of the mouse cortex are labeled by a green fluorescent protein. The study was carried out in accordance with the recommendations in the Guidelines for the Care and Use of Laboratory Animals of the Animal Research Committee. The protocol was approved by the Committee on the Ethics of Animal Experiments. These data sets capture a tomogram with a depth ~1.4 mm from the surface layer of the cortex. The volume data have a size of 512 × 512 × 325 voxels, and the range of capture is 512 × 512 × 1300 *μ*m. Neurons are included within the image, and the direction from the deep part of the brain toward the brain surface is assigned as the positive *z* axis. Apical dendrites, dendrites, soma, and white matter exist in the positive *z* direction. To reduce noise, a median filter is applied for preprocessing the volume data.

For this experiment, the ratios of the eigenvalues *λ*_1,_*λ*_2_, and *λ*_3_ of the Hessian matrix and the eigenvector ***e***_3_ belonging to the third eigenvalue of the Hessian matrix are used to express the shape feature. The feature value *λ*_*ratio*_, which highlights linear structures, is calculated from *λ*_1,_*λ*_2,_ and *λ*_3_ based on

9 $$ {\lambda}_{ratio}=\left\{\begin{array}{c}\hfill {\lambda}_2/{\lambda}_1-{\lambda}_3/{\lambda}_1\hfill \\ {}\hfill {\lambda}_2/{\lambda}_1\hfill \\ {}\hfill 0\hfill \end{array}\right.\begin{array}{c}\hfill \left({\lambda}_1\le {\lambda}_2\le {\lambda}_3\le 0\right)\hfill \\ {}\hfill \left({\lambda}_1\le {\lambda}_2\le 0,{\lambda}_3>0\right)\hfill \\ {}\hfill otherwise\hfill \end{array} $$

Voxels with large *λ*_2_/*λ*_1_ ratios are linear or spherical local structures, whereas those with large *λ*_3_/*λ*_1_ ratios are spherical local structures. As such, voxels with a large difference value for *λ*_2_/*λ*_1−_*λ*_3_/*λ*_1_ are linear local structures. In addition, linear structures with an intensity less than that of the background have positive values of *λ*_1_ and *λ*_2_, whereas those with an intensity greater than that of the background have negative *λ*_1_ and *λ*_2_ values. Because the linear structures included in the target data of the present experiment have an intensity value greater than that of the background, *λ*_*ratio*_ is prevented from becoming increased (in voxels) with an intensity less than that of the background by using the conditions *λ*_1_ ≤ *λ*_2_ ≤ *λ*_3_ ≤ 0 and *λ*_1_ ≤ *λ*_2_ ≤ 0, *λ*_3_ > 0. The feature value ***e′***_3_, which corrects the eigenvector ***e***_3_, is given as

10 $$ \boldsymbol{e}{\boldsymbol{\hbox{'}}}_3=\left\{\begin{array}{c}\hfill {\boldsymbol{e}}_{\boldsymbol{3}}\hfill \\ {}\hfill 0\hfill \end{array}\right.\kern0.5em \begin{array}{c}\hfill \left({\lambda}_1\le {\lambda}_2\le 0\right)\hfill \\ {}\hfill (otherwise)\hfill \end{array} $$

As with ***e′***_3_ and *λ*_*ratio*_, and by using the condition *λ*_1_ ≤ *λ*_2_ ≤ 0, ***e′***_3_ is prevented from having a value in voxels with an intensity less than that of the background.

### White matter

**Figure 5:**
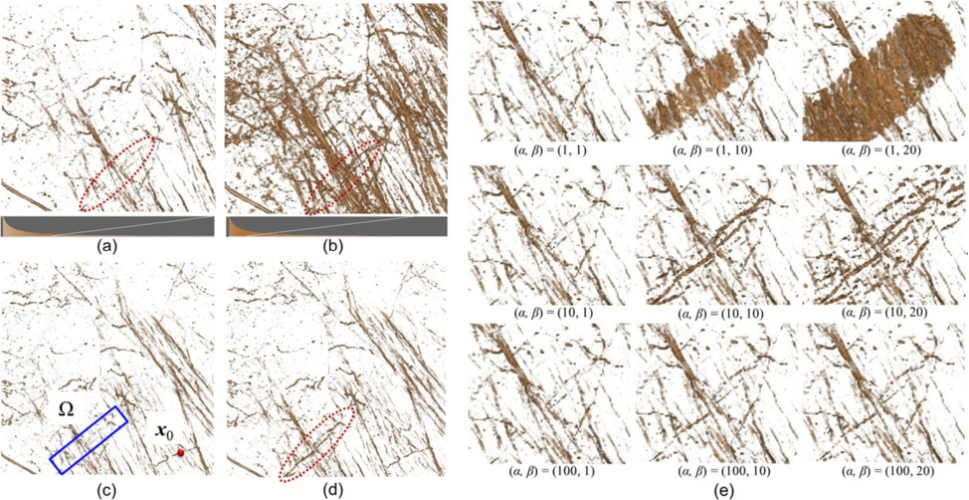
**Interactive visual exploration for overlapping white matter structures.** Visualization results obtained using **(a)** and **(b)** 1D TF presets, **(c)** ***x***_0_ and Ω set by the user, **(d)** the locally refined result and **(e)** effect of the configurable parameters *α* and *β*, wherein the sensitivity and locality of the exploration can be interactively adjusted based on user objectives.

During the experiment, the region containing white matter is trimmed, and ***ν***_*eye*_ at the time of rendering is set as the − *z* direction. Figure 5(a) and (b) shows visualization results with 1D TF presets. We are able to confirm that most of the linear structures are included within the image in the (*x*, *y*, *z*) = (1, 1, 0) direction, but linear structures in directions other than (1, 1, 0) also exist but cannot be visualized clearly. Here, the focus is on linear structures in the direction (1, −1, 0) within the red circle. In the visualization of Figure 5(a), many parts of the linear structures in the red frame have become transparent. In Figure 5(b), linear structures in the (1, 1, 0) direction occlude the structures within the red frame, making it difficult to distinguish them. We attempt to selectively visualize based on the orientation of the linear structures by using ***e′***_3_ as the feature. Because ***e′***_3_ represents the local orientation of the structure, it is better to use the inner product rather than the distance when calculating the dissimilarity *d*_*F*_. The expression for *d*_*F*_ in this case is given as

11 $$ {d}_F\left(\boldsymbol{x},{\boldsymbol{x}}_0\right)=1-\left|\boldsymbol{e}{\hbox{'}}_3\left(\boldsymbol{x}\right)\cdot \boldsymbol{e}{\hbox{'}}_3\left({\boldsymbol{x}}_0\right)\right|. $$

The *TF*_*preset*_ used in Figure 5(a) is used for our method, and the structure already visualized is indicated as a feature point. The ***x***_0_ and Ω are set as shown in (c). The results of the interactive visual exploration with *α* = 10 and *β* = 6 are shown in (d). Compared with the visualization results obtained with 1D TF presets (Figure 5(a) and (b)), the results obtained with our method (Figure 5(d)) present the existence of structures within the red frame that are easily distinguished by users

**Figure 6:**
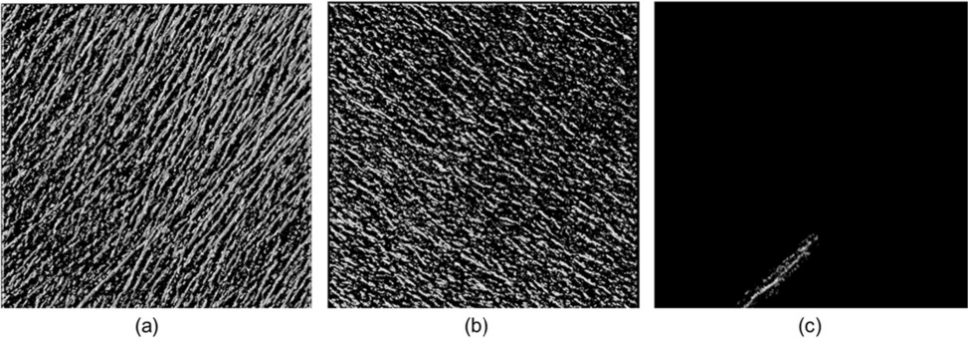
**Three-dimensional TF shift distribution globally computed based on (a) the orientation of the structures, (b) vertically overlapping structures and (c) locally selected by the user.**

We now qualitatively examine the process of visualization by the proposed method by first discussing the validity of the calculated *d*_*F*_. The value of the feature at ***x***_0_ is ***e′***_3_(***x***_0_) = (−0.847, 0.527, 0.0650). The results of the visualization of all voxels having the dissimilarity of the feature with *d*_*F*_ ≤ 0.5 are shown in Figure 6(a). The structures visualized in (a) are those in which the angle with ***e′***_3_(***x***_0_) is within 60°. Figure 6(b) displays the vector ***e′***_3⊥_(***x***_0_), which is perpendicular to ***e′***_3_(***x***_0_) in the *xy* plane, showing the visualization results of all voxels with *d*_*F*_ ≤ 0.5, and structures in which the angle with ***e′***_3⊥_(***x***_0_) is within 60° are visualized. By calculating *d*_*F*_ with the feature 60°, we find that each linear structure in a different direction can be separated. With the proposed method, the visualized area is limited by visualizing structures only within Ω after separating the visualization target structures from other structures by using *d*_*F*_. Figure 6(c) shows the shift Δ of the TF when the proposed method is tested with *α* = 10 and *β* = 6. Structures around the computer mouse cursor are visualized during the operation. Users can use the visualized structures as a guide and can interactively specify these structures targeted for visualization. We see that only TFs of the target structures can be modified by using *d*_*F*_ within the visualized region Ω. Furthermore, the average frames per second for visualization is 53, confirming that the proposed method is interactive in practice.

Next, we examine the effect of the sensitivity *α* and the radius *β* on the visualization results. The parameters are varied so that *α* = 1, 10, and 100 and *β* = 1, 10, and 20, and the results of visualization by combining each of these values are shown in Figure 5(e). When *α* = 1, structures are completely visualized except for the background; when *α* = 10, linear structures in the (1, −1, 0) direction are visualized; and when *α* = 100, the visualization results are the same before and after the operation. When the focus is on visualizing similar structures of ***x***_0_, a suitable parameter value for *α* which enables visualization of linear structures in the direction (1, −1, 0) is *α* = 10. When *α* is larger than this value, it is not possible to visualize targeted structures, and when *α* is smaller than this value, structures outside of those targeted are visualized. Next, when *β* = 1, the visualization results before and after the operation are practically unchanged. When *β* = 10, both the target structures and surrounding structures are visualized, and when *β* = 20, structures corresponding to a wider range than *β* = 10 are visualized. Thus, to visualize the target structures only, it is best to use the smallest *β* possible. However, if *β* is too small, the function cannot be used as a guide when specifying the target structures. Conversely, although it is easy to specify target structures if *β* is large, it is possible that structures outside of those targeted may also be visualized.

As such, it is necessary to select suitable parameters based on user objectives and on the actual situation when attempting to obtain good visualization results. Although the parameters are modulated based on a trial-and-error process, the following guidelines can be considered. Because *α* controls sensitivity of the features that are to be visualized, large *α* values are first suitable to roughly visualize local structures with a variety of features, and then smaller values can be tried to strictly extract the target features. *β* controls the size of the direct editing pointer that defines the minimal region of interest (ROI). Therefore, *β* can be adjusted based on the size of the target structures. Larger *β* is useful when searching non-visualized, transparent structures existing within the volume data. After visualizing a part of the target structure, a small *β* is useful for fine adjustment of the visualization results.

### Dendrites

**Figure 7:**
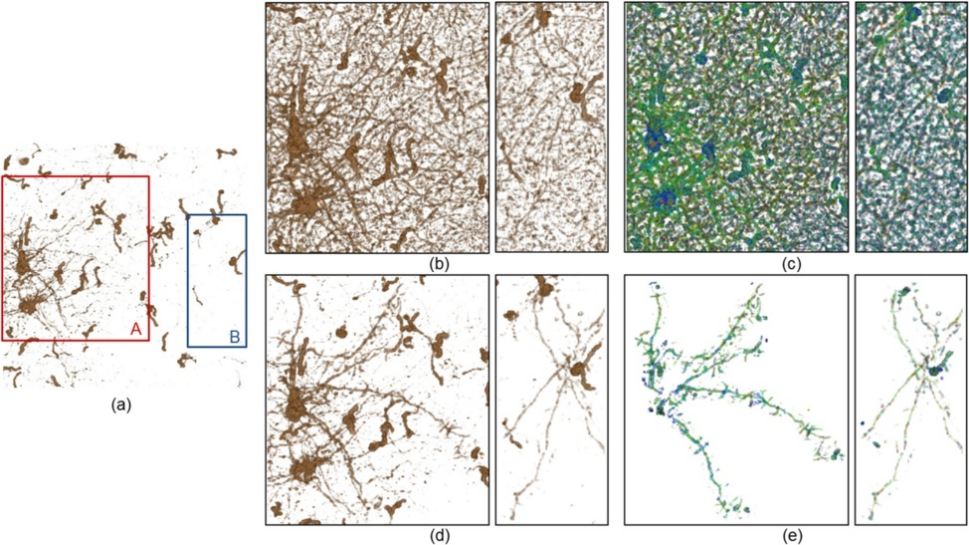
**Comparison between the vessel measure based TF shift** [27] **and the proposed interactive exploration. (a)** 1D TF preset visualization, **(b)** close-up visualization of the area A and B shifted by the vesselness measure, **(c)** the TF shift applied to the area, **(d)** interactive editing results by the proposed framework for tracing specific dendric structures with lower intensity and **(e)** the TF shift.

Next, we apply this method to the area with dendric structures. The eye direction (***ν***_*eye*_) during volume rendering is set as + *z*. Figure 7(a) shows volume rendering results obtained with 1D TFs, which fails to visualize thin dendric structures with lower intensities. Here, we focus on visual exploration of the local area A and B. Figure 7(b) is visualized using previous method, in which we modify the TFs of the area based on the *vesselness* measure used by Lathen et al. [27] for the feature. Linear structures of low intensity appear and run over the *xy* plane around the dendrites, but only some of the structures can be distinguished. Because all linear structures in the data are highlighted as shown in Figure 7(c), it is hard to present the endpoints of one linear structure in a form visible to the eye. By applying the proposed method to this dendritic region, we attempt to observe structures in detail. Equation (10) and *λ*_*ratio*_ are used for the feature to highlight the linear structures. The expression used in this experiment is given:

12 $$ {d}_F\left(\boldsymbol{x},{\boldsymbol{x}}_0\right)=\frac{1}{\sigma}\sqrt{{\left({\lambda}_{ratio}\left(\boldsymbol{x}\right)-{\lambda}_{ratio}\left({\boldsymbol{x}}_0\right)\right)}^2} $$

where the quantity σ is the standard deviation of *λ*_*ratio*_.

The point of the linear structures is denoted as ***x***_0_. To apply small modifications to the visualization results, we use (*α*, *β*) = (0.30, 6). By changing the specified region of Ω, we interactively obtain the visualization result. First, clear structures are visualized among the linear structures when the dendrites within the area B are taken as the starting point. The proposed method enables the presentation of plural structures in forms distinguishable to the eye, which are difficult to observe in semitransparency with methods developed to date. In addition, when using the *vesselness* measure based TF shift, many overlapping structures are displayed simultaneously. However, the proposed method allows the structures of interest to be traced easily on the rendered image. Figure 7(d) shows the visualization results after interactive editing of the dendric structures. The TF shift is shown in Figure 7(e). Comparing the two methods, linear structures that are largely transparent and cannot be distinguished can be visualized using the proposed method.

### Apical dendrites

**Figure 8:**
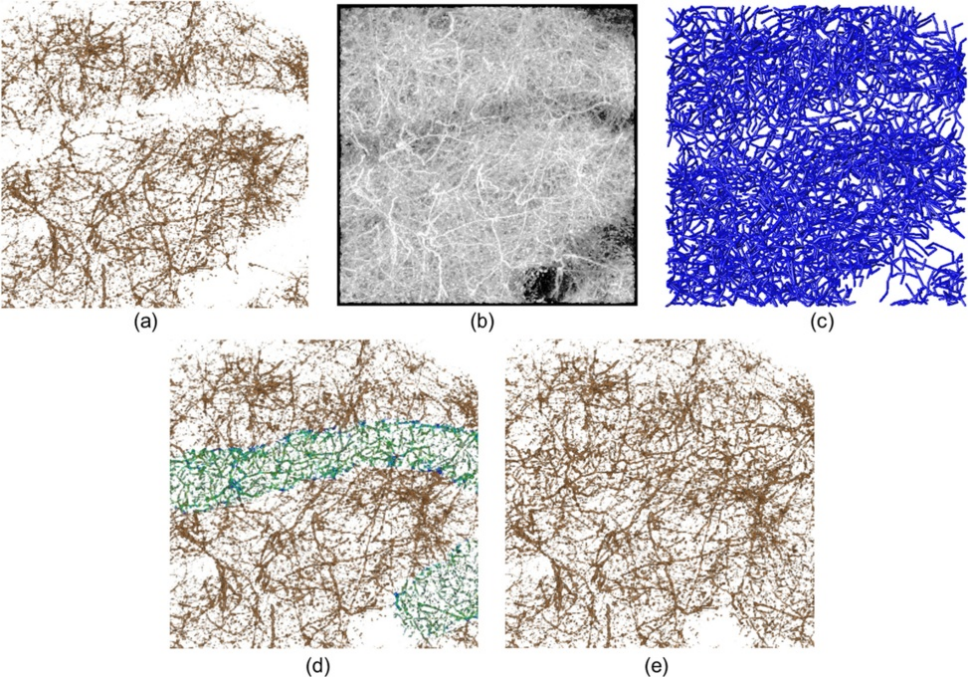
**Apical dendric region represented using (a) TF preset visualization, (b) MIP-based DVR, (c) surface reconstruction by neuron tracing, (d) TF shift applied to the low contrast area locally and interactively and (e) the improved DVR result, which clarifies the connectivity of neural structures.**

Finally, a visualization experiment targeting the apical dendritic region is performed. We applied our data to the Vaa3D visualization software employing virtual finger (VF) techniques [7,8] and compared the visualization results. We tried the visualization functions and the tracing tool in the Vaa3D software, and finally decided to focus on the two functions of MIP (maximum intensity projection) based direct volume rendering (DVR) and Vaa3D-Neuron2 auto-tracing for comparison with our local TF shift techniques. Our visualization targets are the dendric structures possessing a lower intensity located under the blood vessels. Although there are linear dendric structures that cross the blood vessels in Figure 8(a), their connectivity and distribution are not clear with the 1D preset TF.

Figure 8(b) shows the MIP-based DVR results in the Vaa3D software. This rendering mode is effective for visualizing occluded vessels, and some neural structures can actually be observed in the lower intensity area. However, because of a widening in the range of opaque intensity, optical noise or unfocused surrounding structures are also visualized globally, which can decrease the contrast between the neural structures and background areas. This continues to make it difficult to clearly visualize connectivity of the neural structures. Figure 8(c) shows the Vaa3D-Neuron2 auto-tracing result with an auto-thresholding mode [8]. Neural structures are reconstructed as surface models by correcting imperfect parts in the traced areas. However, this function can yield many artifacts, especially around complex crossing structures with low-contrast intensity values. When higher threshold values are used, the artifacts can be reduced but reconstruction of the neural structures in low intensity area is not achieved. This results in a difference in the spatial density of neural structures in low-intensity areas and in other areas. We also note that the tracing result is represented using the reconstructed surface models. Locally improved DVR is not achieved using only vessel tracing tools.

With the proposed method, the TF shift is applied to the low-intensity area locally and interactively. The parameters used in this experiment are (*α*, *β*) = (0.45, 20). Figure 8(d) shows the spatial relationship between the TF shift applied to a low-intensity area and other dendric structures with high intensity. Figure 8(e) is the final rendering result, and the connectivity of the dendric structures can be viewed thanks to locally improved DVR images. Compared with (a), (b) and (c) of Figure 8, the visual appearance of the spatial density for vessel structures is corrected, which generates a more natural visualization result in (e). Thus, our approach can simultaneously visualize regions that are below blood vessels without affecting regions that are not.

## Discussion

So far, most interaction techniques [7,8,32] assume that the entire shapes of the target structures is first visualized in the screen. Then, by pinpointing a 3D position on the rendered image, the user can extract a part of the 3D structure to specify his/her ROI. However, microscopic images obtained from brain tissues sometimes contain a variety of neural structures with complex crossings and overlapping linear structures. In these cases, even if the 3D location is specified, the tracing task is difficult and often fails to extract a single linear structure because undesired surrounding structures are simultaneously selected. In the developed software, we can start the tracing task on the partially-visualized structure and can expand it to transparent regions with low-intensity areas where no structures are yet visualized (see Figure 3). In addition, thanks to the multidimensional TF, the user can limit the visible structures in a robust way based upon a combination of 3D features such as direction, radius and textures (Figures 6, 7 and 8). The multidimensional TF design and its combination with the direct editing interface are main contributions of this work in biological visualization. In addition, three-dimensional images with scalar intensity values measured from electron microscopes and confocal microscopes as well as CT/MRI images can be applied to our software. We will further study practical application to other types of volume data

The automation of transfer function (TF) generation is a critical issue in volume rendering. Specifically, in biological volume visualization, users often focus on overlapped neural structures or internal structures occluded by other surrounding tissues. When automatic TF tuning [26,27] is applied globally, many structures are simultaneously visualized and some of these structures occlude the target structures. In Figure 7(c), because similar structures in the data are highlighted, it is difficult to present the endpoints of one linear structure in a visually distinguishable form. Applying machine learning or clustering approaches [28] are interesting methods to automate TF configuration, but they require prior knowledge to learn the target structures.

Knowledge exploration for newly-measured data is regarded as a user-dependent problem in many situations. The advantage of the interactive, manual TF configuration is that features of interest can be locally explored based on the user's preferences. This user-dependent localization of information visualization is an important factor to transfer the user's biological knowledge into the visualization system. Also, because the developed system does not require any time-consuming setup, it can provide a practical environment for rapid visualization of the measured data. Combining the interactive localization with automatic TF tuning would be interesting and has the possibility of a more intelligent interface for volume visualization, and we would like to further study this on the semi-automatic framework.

## Conclusions

In this study, a method is proposed for interactive and local improvement of visualization by volume rendering in two-photon microscope images containing regions in which linear nerve structures crisscross in a complex manner. For this method, multidimensional features and interactive methods are used for selectively visualizing structures. Users can specify the structures and regions of interest on the rendered image and only visualize similar structures in the regions of interest. The proposed method introduces the parameters *α* and *β*, which are freely tuned by the user. The sensitivity *α* determines how easy it is to change visualization results by similarity, whereas the radius *β* determines the size of the region of interest.

By applying the proposed method to an image of a mouse brain acquired by two-photon microscopy, we visualize white matter, dendrites, and apical dendrites in which we verify the proposed method in an experiment in the region containing white matter. The proposed method is characterized by multidimensional features and interactive methods that are effective for visualizing target structures. Furthermore, we investigate the effect of parameters *α* and *β* on the results of visualization, where we find that a large *β* is suitable for search purposes and a smaller *β* is effective for small modifications of the visualization results. Because the suitable values of *α* and *β* depend on the situation, the proposed method promises that visualization can be implemented with flexibility, and that the parameters can be determined by the user to suit their particular visualization requirements.

### Availability of supporting data

The data sets supporting the results of this article are included within the article.
